# Pancreatic Duct Cells Isolated From Canines Differentiate Into Beta-Like Pancreatic Islet Cells

**DOI:** 10.3389/fvets.2021.771196

**Published:** 2022-01-05

**Authors:** Yuhua Gao, Weijun Guan, Chunyu Bai

**Affiliations:** ^1^Institute of Precision Medicine, Jining Medical University, Jining, China; ^2^Institute of Animal Sciences, Chinese Academy of Agricultural Sciences, Beijing, China

**Keywords:** canine (dog), diabetes mellitus, pancreatic duct cells, islet beta cells, differentiation

## Abstract

In this study, we isolated and cultured pancreatic ductal cells from canines and revealed the possibility for using them to differentiate into functional pancreatic beta cells *in vitro*. Passaged pancreatic ductal cells were induced to differentiate into beta-like pancreatic islet cells using a mixture of induced factors. Differentiated pancreatic ductal cells were analyzed based on intracellular insulin granules using transmission electron microscopy, the expression of insulin and glucagon using immunofluorescence, and glucose-stimulated insulin secretion using ELISA. Our data revealed that differentiated pancreatic ductal cells not only expressed insulin and glucagon but also synthesized insulin granules and secreted insulin at different glucose concentrations. Our study might assist in the development of effective cell therapies for the treatment of type 1 diabetes mellitus in dogs.

## Introduction

Diabetes mellitus (DM) is a commonly encountered disease in veterinary practice and is caused by absolute or relative insulin deficiency (1, 2). Pancreatic beta cells have well-tuned machinery to sense glucose and secrete insulin to control glucose homeostasis; therefore, the transplantation of beta cells as a new cure for DM has become an attractive therapeutic strategy, but its application has limitations based on the donor cell source (3, 4). In the previously study, the pancreatic beta cells had been obtained from numerous stem cells in human, monkey, mice, pig, chicken, including embryonic stem cells (ESCs), induced pluripotent stem cells (iPSCs), and adult stem cells (5–8). However, no reports about differentiation of beta cells from pancreatic ductal cells in canine. Pancreatic ductal cells are a one of stem cells in the pancreas that are responsible for the formation of the pancreas from the embryo to fetal period, and these can be induced to differentiate into pancreatic endocrine cells and exocrine cells *in vivo* and *in vitro* when provided with different treatments. In this study, we report that CK19-postive ductal cells can be isolated from the pancreas of canines and directly induced to differentiate into beta-like islet cells. This research provides a novel source for the transplantation of beta cells and establishes a model to explore pancreatic beta cell neogenesis *in vitro* and *in vivo* in companion animals.

## Materials and Methods

### Ethics Statement

The five dying canine newborn (gestational age 68 day) were selected which cesarean section performed because of dystocia in the Experimental Base Institute of Animal Sciences (Beijing), Chinese Academy of Agricultural Sciences. All animal procedures were approved by the Institutional Animal Care and Use Committee of the Chinese Academy of Agricultural Sciences and Jining Medical University (2019-JZ-001).

### Immunolocalization of Pancreatic Ductal Cells in the Canine Pancreas

The pancreatic tissues from one newborn pups were fixed in 4% paraformaldehyde (PFA) overnight at 4°C, embedded in paraffin, and sectioned for 5 μm thickness. Sections of embedded pancreatic tissue were deparaffinized and rehydrated, and microwave antigen retrieval with citric acid buffer (PH6.0) and sections were incubated with 5% bovine serum albumin (BSA) for 30 min at room temperature. Primary polyclonal antibodies (anti-insulin, Bioss, bs-0862R, 1:100, and anti-CK19, Abcam, ab52625, 1:100, ALK3, Bioss, bs-25048R, 1:100) were incubated with the sections overnight at 4°C. Sections were subsequently incubated with FITC- or Cy5-conjugated secondary antibodies (ABclonal technology, Wuhan, China) (1:100) for 2 h at room temperature. The primary antibodies were replaced by PBS using a negative control. DAPI was used to stain the nucleus.

### Isolation and Culture of Pancreatic Ductal Cells

The whole pancreas from four newborn pups was washed three times with PBS buffer, cut in to about 1 mm^3^ small pieces, and then digested with 1 mg/mL collagenase P (Sigma–Aldrich, USA) for 30 min at 37°C, respectively. The digested suspension was neutralized with fetal bovine serum (FBS, PAN Biotech), filtered through 80 μm cell strainers, and centrifuged at 1,000 × g for 10 min. The 5 × 10^6^ cell suspension were seeded into 6 cm cell culture dish and cultured in RPMI 1640 (Gibco, USA, 11.11 mM Glucose) supplemented with 10% FBS, 10 ng/ml recombinant human bFGF, and 10 ng/ml recombinant human EGF (Peprotech, USA) at 37°C, 5% CO_2_ for 48 h. Then the suspended cells, which containing pancreatic acinar cells and pancreatic islet cells, were removed, and the adherent cells, which containing pancreatic ductal cells, fibroblast, and mesenchymal cell, were continued to culture. When the cells reached 80–90% confluence, 0.25% trypsin and 0.01% EDTA (Gibco, USA) was added to dissociate the cells from cell dish, and the digested suspension was neutralized with FBS. The collected cells were wished with phosphate buffer saline (PBS) supplemented with 1% BSA for three times, and then the cells were incubated with PBS supplemented with 4% BSA for 10 min. ALK3 (as known as BMPR1A) is a surface marker for pancreatic ductal cells, which has been reports in previously study, and co-positive expression in pancreatic ductal cells with CK 19 (9). Therefore, the rabbit monoclonal to ALK3 antibody (Bioss, China, bs-25048R, 1:100) were added in cell suspension for incubation for 1 h at room temperature, then cells were wished with phosphate buffer saline (PBS) supplemented with 1% BSA for three times, FITC connected goat anti-rabbit second antibody (1:100, Zhongshan Golden Bridge, Beijing) was added into cells and incubated for 1 h at room temperature in darkroom. The cell were re-suspend in 500 μL PBS after removed the second antibody, and the positive cells were purified using flow cytometry (Beckman Coulter MoFlo XDP, USA). They were then seeded onto 6-well cell culture plates and cultured in H-DMEM (Gibco, without sodium pyruvate, containing 50% F12 medium, 17.5 mM glucose) supplemented with 10% FBS, 10 ng/ml recombinant human bFGF, and 10 ng/ml recombinant human EGF. The positive rate of CK 19 were detected using CK 19 antibody with flow cytometry in the expanded cells. The rabbit monoclonal to CK 19 antibody (1:100) were added in cell suspension, and PE connected goat anti-rabbit second antibody (1:100, Zhongshan Golden Bridge, Beijing) was used to analyze the positive cells.

For analyzing the expression of PDX 1 and CK 19 in different passages, the pancreatic ductal cells from passage 3, passage 9, and passage 15 were detected using immunofluorescence. The cells were seeded on glass coverslips, fixed by 4% paraformaldehyde, permeabilized by 0.25% Triton X-100, and then incubated with 10% goat serum. The rabbit monoclonal to CK 19 antibody (1:200) and the mouse monoclonal to PDX 1 antibody (1:200) were incubated with these cells overnight at 4°C. Coralite594 connected goat anti-mouse second antibody (1:200, Proteintech, China, SA00013) and TRITC connected goat anti-rabbit second antibody (1:200, Proteintech, China, SA0007) were added in these cells as a fluorescence signal. The images were acquired using a laser-scanning confocal microscope (Leica TCS-SP8 SR), and then the intensity of immunofluorescence were calculated with Image J tools.

### Growth Dynamics

To detect growth dynamics, pancreatic ductal cells from different passages were seeded in triplicate onto 24-well cell culture plates at a density of 5 × 10^4^ cells/well, and the cells were counted every day for 7 days. The population doubling time (PDT) was calculated as previously reported (10).

### Detection of Markers in Pancreatic Ductal Cells and Differentiated Pancreatic Islet Cells

Pancreatic ductal cells were seeded on glass coverslips, fixed in 4% PFA for 15 min, and permeabilized with 1% triton X-100 for 10 min. They were then blocked in 4% BSA for 30 min at room temperature and incubated with primary antibodies (anti-CK19, Abcam, 1:200; PDX 1, Abcam, 1:100, anti-insulin, Abcam, 1:200; anti-glucagon, CST, 1:200) at 4°C overnight. Next, the cells were incubated with secondary antibodies conjugated with FITC or Cy5. The primary antibodies were replaced by PBS using a negative control. Positive cells were observed using a Leica TCS-SP8 SR confocal microscope.

### Differentiation of Pancreatic Ductal Cells Into Beta-Like Pancreatic Islet Cells

To differentiate pancreatic ductal cells into beta-like pancreatic islet cells, the method from previously reported was used in our study (11), 5 × 10^5^ cells were seeded into 6-well cell culture plates and cultured in DMEM/F12 containing 10 nM exendin-4, 100 pM recombinant human hepatocyte growth factor (HGF), 2 nM activin A, and 10 mM nicotinamide (Peprotech, USA) thus culture medium was changed every 2 days for 14 days. Insulin and glucagon levels were detected using immunofluorescence. Randomly selected 10 non-overlapping visual fields were observed and photographed to calculated positive ratios.

### Real Time PCR

The total RNA from pancreatic ductal cells and differentiated cells were extracted with Trizol reagent (Invitrogen, USA) and reverse transcribed using HiFiScript cDNA Synthesis Kit (CWBIO, China). PCR was performed using MagicSYBR Mixture (CWBIO, China) with special primers. Insulin, F: GAGCCTTCGTTAACCAGCACCTG, R: GGCCTTAGGCGTGTAGAAGAAGC;SOX9,F: TCTGGAGGCTGCTGAACGAGAG, R:TGTAATCCGGGTGGTCTTTCTTGTG; NKX2-2,F: TGTCAGCCGTCTTCTAAAGCAAGTG, R:AGAGCGAAATCTGTCACCAGTTGTC; NGN3, F: CCAAGATCGAGACGCTACGCTTC, R: TAGAGGCTGTGGTCCGCTATGC. Gene expression was detected on the Light Cycler 480 PCR system. Each experiment was performed in duplicate in 96-well plates and repeated three times, relative expression was calculated using the 2^−ΔΔ^Ct method.

### Glucose-Stimulated Insulin Secretion in Beta-Like Pancreatic Islet Cells

The 1 × 10^4^ cells were seeded into 24-well cell culture plate, changed to induced medium after 24 h, and then cultured for 14 day continuously to obtain beta-like pancreatic islet cells. To determine whether insulin release from beta-like pancreatic islet cells was glucose-dependent, we used 2 and 20 mM glucose to treat the beta-like pancreatic islet cells and measured insulin release with a dog insulin ELISA kit (Boyan Biotech, China) in accordance with a previous report (12, 13). Briefly, the cells were washed three times with 200 μL Krebs buffer for 5 min each time, and were pre-incubated in 200 μL 2 mM glucose Krebs buffer per well for 2 h to remove residual insulin. The cells were washed three times with Krebs buffer, and incubated in 200 μL 2 mM glucose Krebs buffer per well for 30 min, the supernatant were collected. Then cells were washed three times with Krebs buffer, and incubated in 200 μL 20 mM glucose Krebs buffer per well for 30 min, the supernatant were collected. This sequence was repeated two additional times. The insulin content of supernatant was measured using ELISA kit according to the manufacturer's instructions, and the plate was scanned by microplate reader (Bio-Rad 680, US). Finally, the cells were dissociated with 0.25% trypsin and 0.01% EDTA from culture plate to count automatically cell number using Countess II FL system (Invitrogen, USA). The insulin concentration were calculated according the cell number per cell culture plate.

### Transmission Electron Microscopy

Beta-like pancreatic islet cells were collected and fixed in pre-cooled 2.5% glutaraldehyde for at least 2 h and then rinse three times in PBS (0.1 mol/L) for 5 min each time. After post-fixation in 1% osmic acid solutions 3 h and sequential dehydration, cells were embedded in EPON812 resins. Ultrathin sections were cut and collected onto grids, stained with uranium and lead citrate, and observed under a Hitachi TEM system (Tokyo, Japan). Intracellular insulin synthesis is a typical feature of pancreatic beta cells, which initially appear as pale gray cores surrounded by a small electron-lucent area under an electron microscope, and then, insulin is packaged into secretory granules. The secretory granules gradually become dark polygonal crystalline cores surrounded by a light halo with the insulin condensing into granules (13–15). The insulin granules were obtained according the above description. Assessment of ultrastructure was performed by a blinded observer.

### Statistical Analysis

Data are presented as the mean ± Standard Error of Mean (SEM). All experiments were performed at least three times, independently and in triplicate. Differences were assessed using the Student's *t*-test (two groups) or one-way ANOVA and *post-hoc* Tukey's honestly significant difference tests (more than 2 groups) unless otherwise noted. All data were analyzed using the GraphPad Prism 6.0 software (GraphPad Software, USA). Statistical significance was defined as ^*^P < 0.05, ^**^P < 0.01, and ^***^P < 0.001.

## Results

### Spatiotemporal Distribution of Pancreatic Ductal Cells in Pancreas

**Figure 1:**
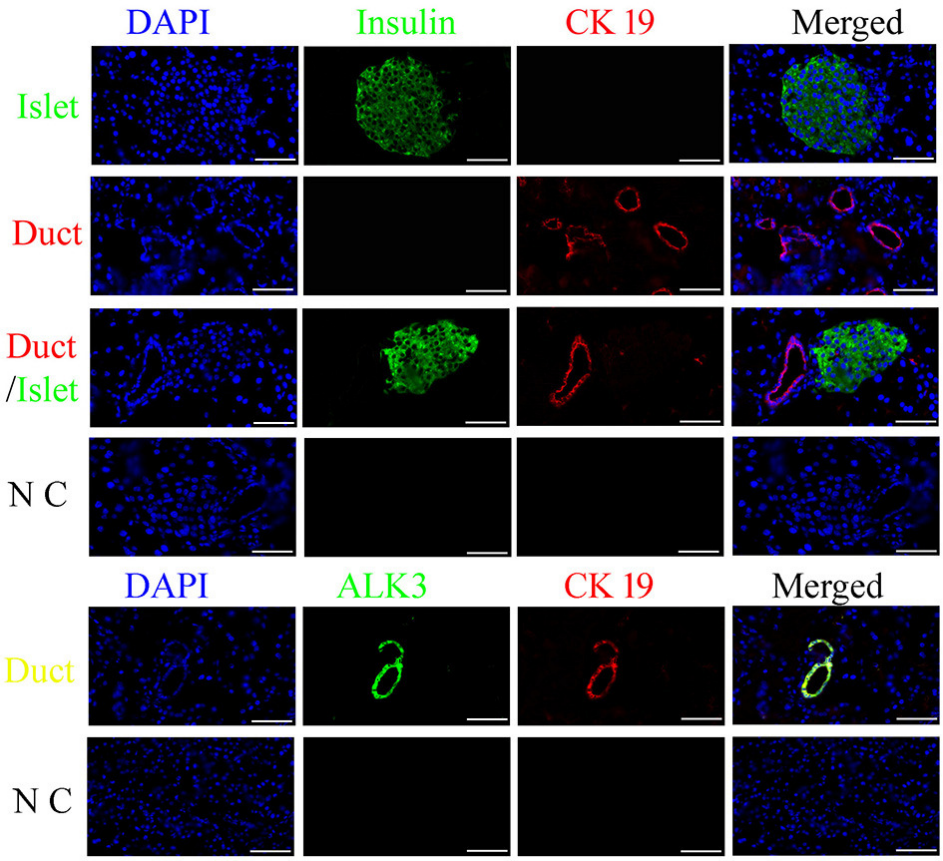
Spatiotemporal distribution of canine pancreatic ductal cells. Insulin (green) is used to locate the pancreatic islets, and CK19 (red) and ALK3 (green) are the marker for duct, which were detected using an antibody with immunofluorescence analysis in the pancreas tissue, nuclear counterstaining with DAPI (blue). CK19 and ALK3 are the main markers for ductal epithelial cells, and CK19 and ALK3-positive cells were organized and formed a tubular structure, near insulin-positive cells. N C: negative control, the primary antibodies were replaced by FBS. Scale bar = 50 μm.

Insulin is usually used to locate pancreatic islets, and CK19 is the main marker for epithelial cells, which were detected using an antibody with immunofluorescence analysis in the canine pancreas. ALK3 (as known as BMPR1A) is a surface marker for pancreatic ductal cells, which has been reports in previously study (9). The results demonstrated that most CK19-positive cells were near insulin-positive cells, well-organized, and formed a tubular structure, and co-expressed with ALK3 in pancreatic ductal cells (Figure 1). These data revealed that similar to that for other organisms, CK19 and ALK3 can be used to locate pancreatic ductal cells in the canine pancreas.

### Isolation and Culture of Pancreatic Ductal Cells

**Figure 2:**
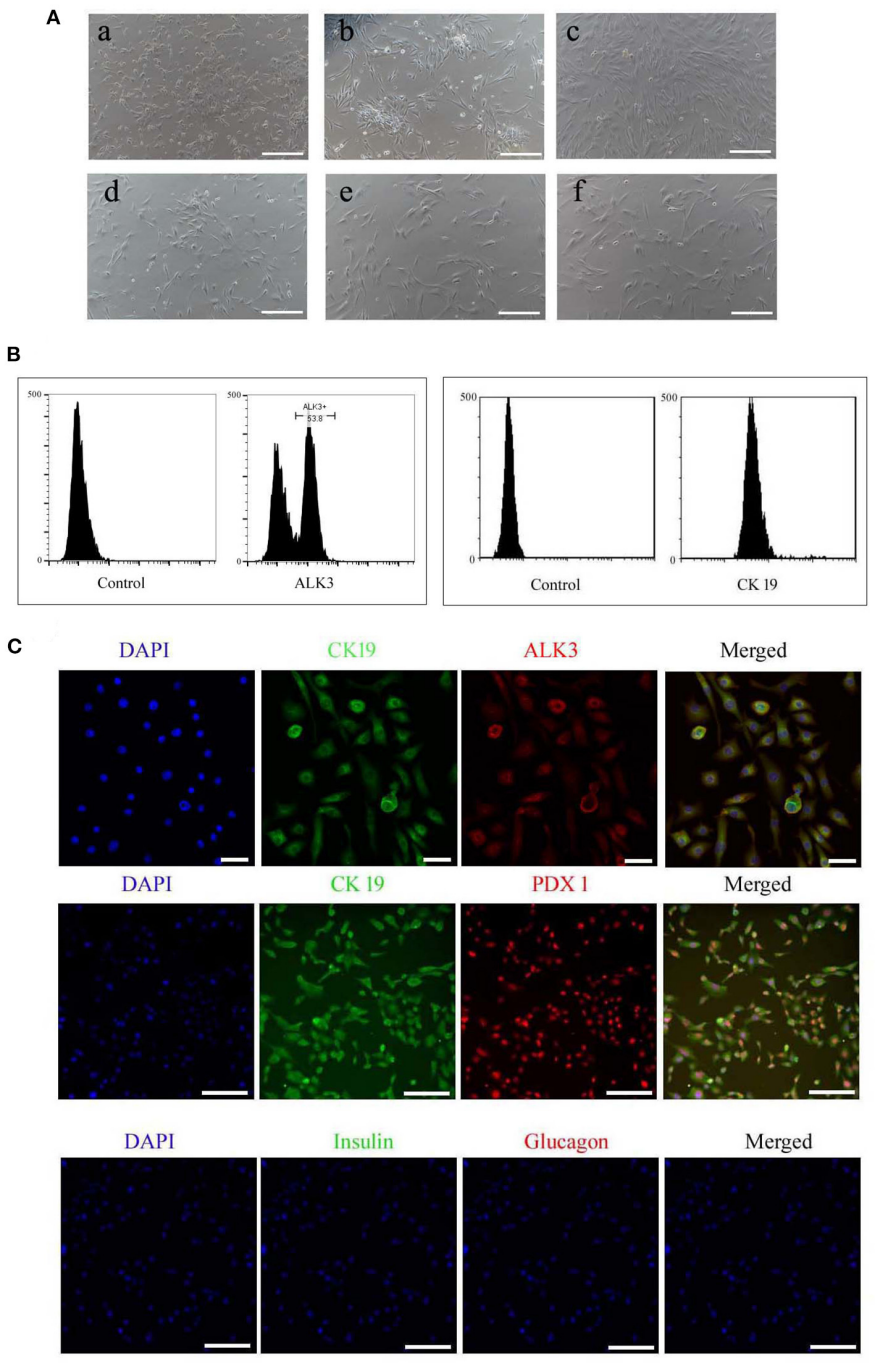
Morphology and specific makers in pancreatic ductal cells. **(A)** Morphology of primary cultured and sub-cultured pancreatic ductal cells *in vitro*. (a) Pancreatic cells plated onto microplates after 72 h. (b) Pancreatic ductal cells were purified by ALK3 antibody using FCM and plated onto culture plates after 72 h. (c) Pancreatic ductal cells before subculture. (d) Pancreatic ductal cells at passage 3. (e) Pancreatic ductal cells at passage 9. (f) Pancreatic ductal cells at passage 15. Scale bar = 100 μm. **(B)** ALK3 positive cells were purified using FCM from primary cells (left penal), the data showed more than 50 percent cells were positive for ALK3 in pancreatic primary cells. CK 19 is a universal marker for pancreatic ductal cells, and then expression of CK 19 were analyzed in the purified cells using FCM (right penal). The data indicated the purified cells were positive for CK 19. **(C)** Detection of CK19, ALK3, and PDX1 by IF staining in purified pancreatic ductal cells. The data demonstrated that PDX1 was located in the nucleus, CK19 was positive in the cytoplasm, ALK 3 was positive in cytomembrane, and insulin and glucagon were negative in pancreatic ductal cells. Scale bar = 50 μm.

The isolated cells adhered and began growing within 72 h, and they were initially round or irregularly shaped (Figure 2A), after 6 days in culture, colonies formed and attained 80% confluence, then were collected using trypsinization. The cell suspension was obtained after enzyme digestion, and the pancreatic ductal cells were purified using the ALK3 antibody with flow cytometry (Figure 2B), the purified cells also positive for CK 19 (Figure 2B). These cells were cultured in cell culture dishes and displayed polygonal, fusiform, and irregular shapes (Figure 2A). Pancreatic and duodenal homeobox 1 (PDX1) is a main marker for pancreatic stem cells, and its expression is maintained during development. In our research, we detected the expression of PDX1 in purified pancreatic ductal cells after three passages expended culture combined with CK19, CK 19, and ALK3 or glucagon and insulin using antibodies, and the results are shown in Figure 2C. PDX1 was located in the nucleus, CK19 was positive in the cytoplasm, ALK 3 was positive in cytomembrane, and insulin and glucagon were negative in pancreatic ductal cells.

**Figure 3:**
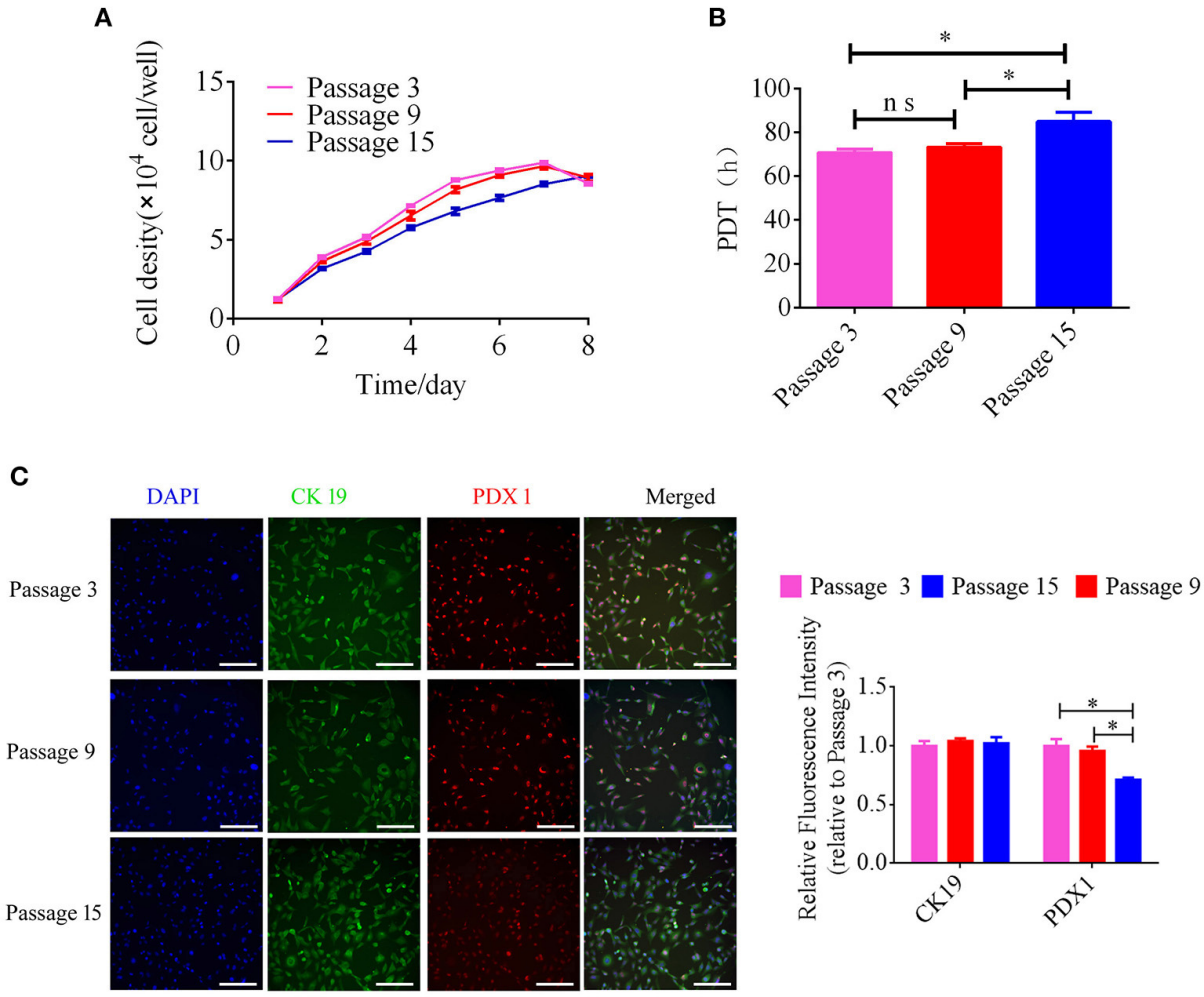
Analysis of growth kinetics and specific makers in pancreatic ductal cells at Passage 3, 9, and 15. **(A)** Growth curves of pancreatic ductal cells cultured at passage 3, 9, and 15. Growth curves had a typical sigmoidal shape, indicating the latent, exponential growth, and stationary phases (*n* = 3). **(B)** Analysis of population doubling time (PDT) in pancreatic ductal cells at passage 3, 9, and 15 (*n* = 3). **(C)** Detection of CK19 and PDX1 by IF staining in pancreatic ductal cells at passage 3, 9, and 15, the expression level of PDX 1 was gradually down-regulated with the extended culture. ^*^P < 0.05.

The growth kinetics of pancreatic ductal cells at passages 3, 9, and 15 were calculated using growth curves, and the growth curves of these cells were typically sigmoidal (Figure 3A). After a latency phase of ~2–3 days, pancreatic ductal cells entered the logarithmic phase, followed by the plateau phase after ~7 days. The PDTs of passage 3, 9, and 15 were 70.83 ± 0.93, 73.28 ± 0.89, and 84.95 ± 2.39 h, respectively (*n* = 3). The average PDT of pancreatic ductal cells was different at passages 3, 9, and 15, as shown in Figure 3B. Our data indicated that pancreatic ductal cells have higher proliferation potential *in vitro*, but this potential decreased with the extension of passages. For analyzed the expression of PDX 1 and CK 19 during culturing *in vitro*, we detected these cells from passage 3, 9, and 15. The results showed in Figure 3C, the relative fluorescence of CK19 were 1.00 ± 0.04, 0.99 ± 0.03, 0.95 ± 0.01 in passage 3, 9, and 15, the PDX1 were 1.00 ± 0.05, 0.98 ± 0.02, 0.76 ± 0.00 in passage 3, 9, and 15, respectively (*n* = 3), and indicated that the expression level of PDX 1 was gradually down-regulated with the extended culture.

### Differentiation of Pancreatic Ductal Cells Into Beta-Like Pancreatic Islet Cells

**Figure 4:**
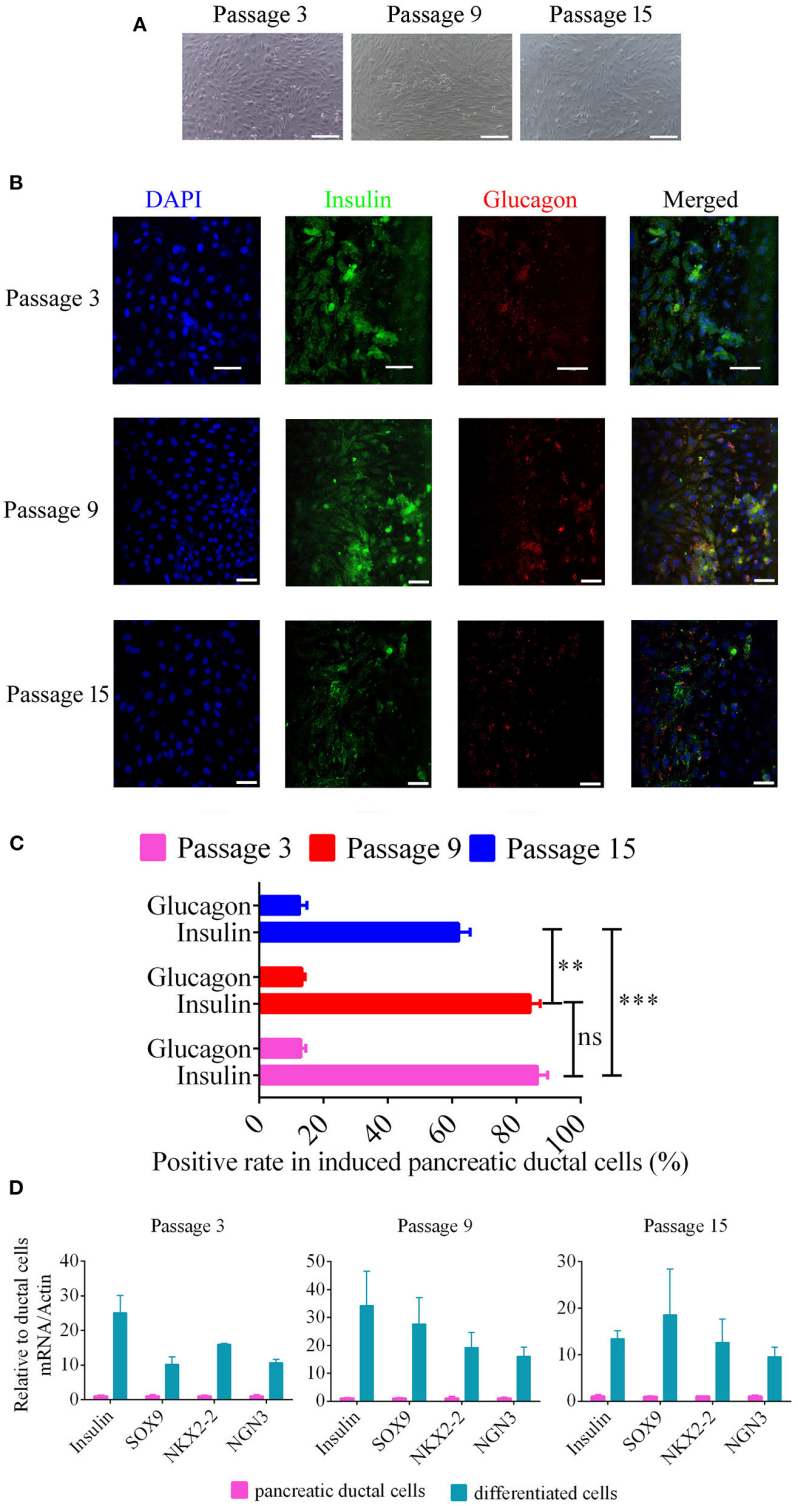
Beta-like pancreatic islet cell differentiation from pancreatic ductal cells. **(A)** Morphology of differentiated ductal cells, cell clusters were appeared after 14 days of continuous treatment. Scale bar = 100 μm. **(B)** The expression of insulin and glucagon were detected using immunofluorescence in induced pancreatic ductal cells derived from different passages. Scale bar = 50 μm. **(C)** Analysis of the rate of insulin and glucagon positivity in induced pancreatic ductal cells derived from different passages (*n* = 10). **(D)** Gene expression of differentiated cells from ductal cells at passage 3, 9, and 15 compared with normal cells. ^**^P < 0.01, ^***^P < 0.001.

To analyze the potential for pancreatic ductal cells to differentiate into pancreatic islet cells, we used a previously reported method (11), which was verified for pancreatic ductal cells from human, mouse, rat, and chicken, to induce canine pancreatic ductal cells from different passages to differentiate into pancreatic islet cells. Cell clusters were appeared after 14 days of continuous treatment (Figure 4A). Insulin expression and secretion are typical characteristics of pancreatic beta cells, and glucagon is a marker of pancreatic alpha cells. Primary antibodies with immunofluorescence were used to analyze the expression of insulin and glucagon in differentiated pancreatic ductal cells after 14 days of continuous treatment. The positive rate of insulin were 86.54 ± 0.99, 84.24 ± 1.00, 62.00 ± 1.20, the positive rate of glucose were 12.91 ± 0.51, 13.25 ± 0.31, 12.50 ± 0.75 in differentiated pancreatic ductal cells from passages 3, 9, and 15, respectively (*n* = 10). The data showed that cells positive for insulin and glucagon were observed among differentiated pancreatic ductal cells from passages 3, 9, and 15, but the rate of positivity gradually declined with the extension of passages (Figures 4B,C). For further demonstrating the differentiation of pancreatic ductal cells, the mRNA levels of Insulin, SOX 9, NKX2-2, and NGN3 were detected by qPCR. The expression level of insulin of differentiated cells from passage 3, 9, and 15 were 25.13 ± 8.67, 34.29 ± 21.22, 13.42 ± 2.99 relative to ductal cells, respectively. The expression level of SOX9 of differentiated cells from passage 3, 9, and 15 were 10.24 ± 3.64, 27.63 ± 16.36, 18.57 ± 17.07 relative to ductal cells respectively. The expression level of NKX2-2 of differentiated cells from passage 3, 9, and 15 were 15.98 ± 0.40, 19.28 ± 9.23, 12.64 ± 8.68 relative to ductal cells, respectively. The expression level of NGN3 of differentiated cells from passage 3, 9, and 15 were 10.66 ± 1.74, 16.01 ± 5.76, 9.55 ± 3.62 relative to ductal cells, respectively. The mRNA level of Insulin, SOX 9, NKX2-2, and NGN3 were dramatically elevated after 14 days of continuous treatment (Figure 4D).

**Figure 5:**
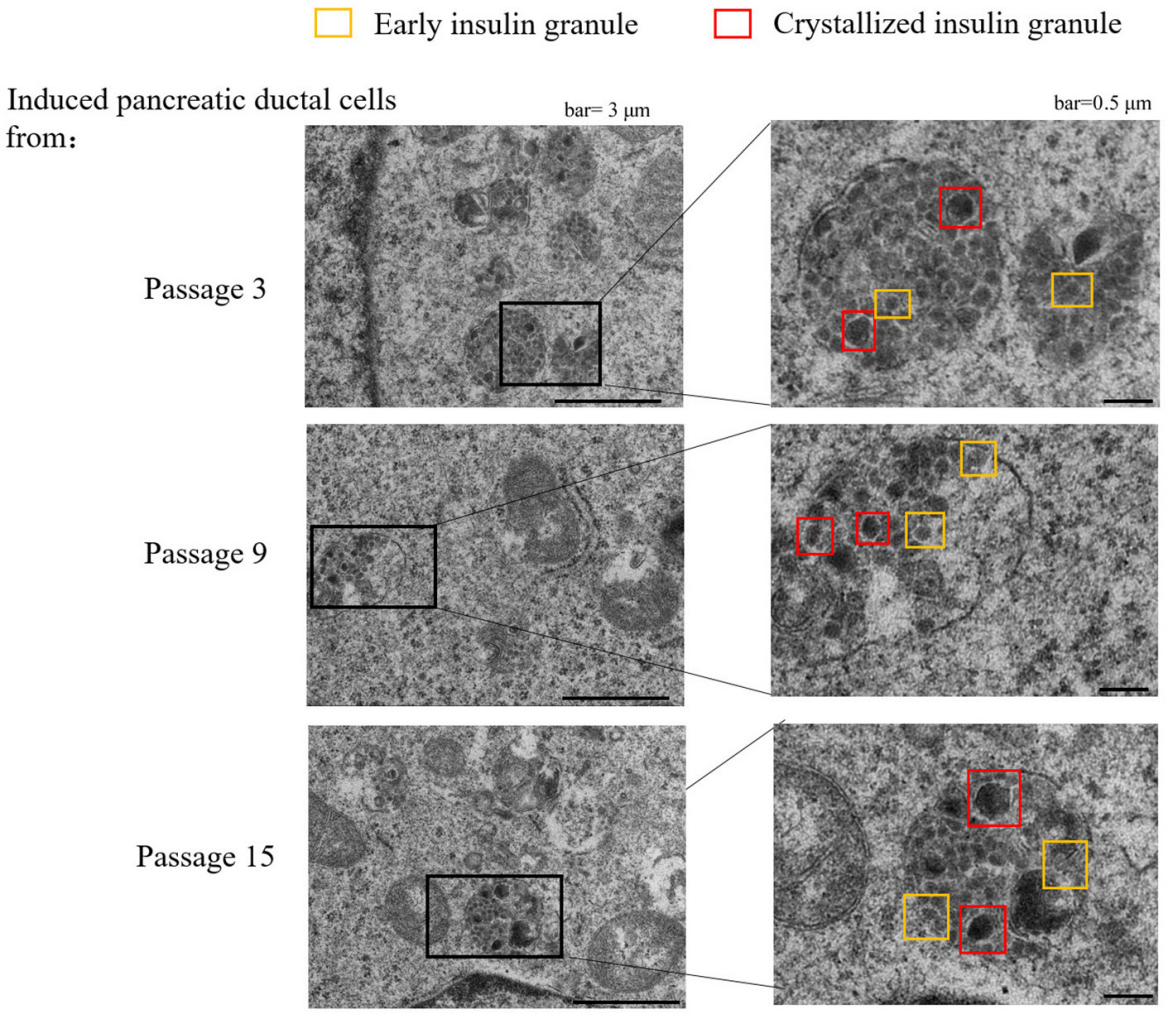
Transmission electron microscope of granules in sectioned cells with representative crystallized insulin granules (red) and early insulin granules (yellow) in induced pancreatic ductal cells derived from different passages.

Insulin granules are a special organelle for pancreatic beta cells, and electron microscopy was used to analyze the granular ultrastructures in beta-like pancreatic islet cells (8, 13). Intracellular insulin synthesis initially appear as pale gray cores surrounded by a small electron-lucent area (Early insulin granules). And then, insulin is packaged into secretory granules, which gradually become dark polygonal crystalline cores surrounded by a light halo with the insulin condensing into granules (Crystallized insulin granules). The crystallized insulin granules can be released to the outside of cells via endocytosis under high-concentration glucose treatment. In this study, insulin granules in differentiated pancreatic ductal cells were detected using transmission electron microscopy, and the crystallized insulin granules and early insulin granules were observed, and the size of these granules were about 0.5 microns in diameter (Figure 5).

**Figure 6:**
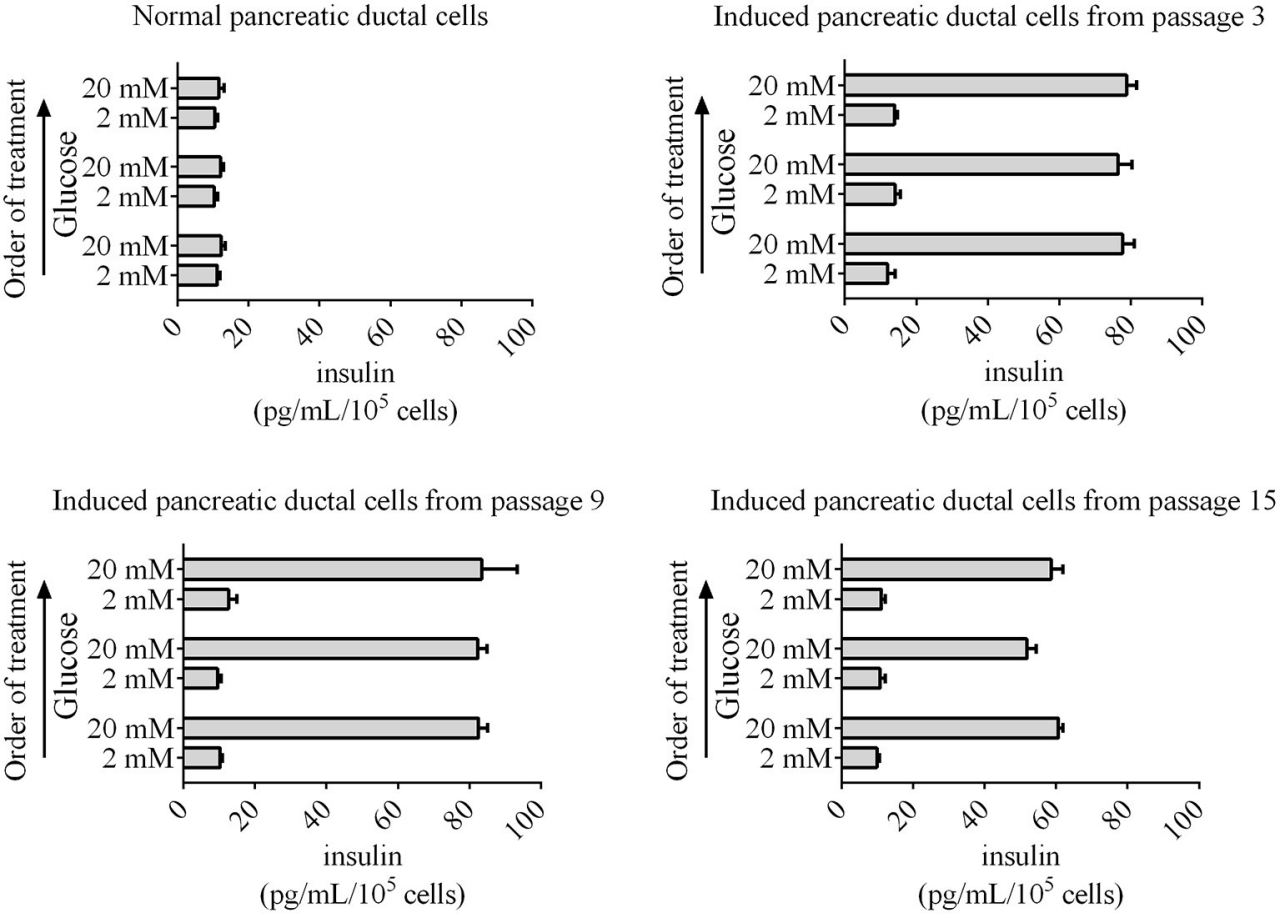
Glucose-stimulated insulin secretion by induced pancreatic ductal cells derived from different passages. An ELISA was used to measure secreted insulin from cells stimulated sequentially with 2, 20, 2, 20, 2, and 20 mM glucose with 30 min of incubation for each concentration. Insulin was secreted from induced pancreatic ductal cells but not normal pancreatic ductal cells (*n* = 3).

Insulin secretion is another characteristic of normal pancreatic beta cells, which can release insulin to the outside of cells according to the glucose concentration. The pancreatic beta cells differentiated from human, mouse, rat, and chicken stem cells also have this capacity for glucose-stimulated insulin secretion. Next, glucose-stimulated insulin secretion from differentiated pancreatic ductal cells was detected using ELISA. Our data revealed that the induced pancreatic ductal cells secreted insulin when treated the glucose with concentration for 2 or 20 mM, whereas the normal pancreatic ductal cells did not secrete insulin (Figure 6). The concentrations of insulin were 77.70 ± 3.20, 76.41 ± 3.99, 78.89 ± 4.80 pg/mL/10^5^ cells after three-times high-concentration glucose treatment in differentiated pancreatic ductal cells from passages 3, respectively, 82.55 ± 4.61, 82.38 ± 2.52, 83.45 ± 9.91 pg/mL/10^5^ cells in cells from passages 9, respectively, 60.64 ± 2.35, 51.83 ± 2.57, 58.67 ± 3.30 pg/mL/10^5^ cells in cells from passages 15, respectively, and 12.28 ± 1.92, 12.18 ± 0.74, 11.7 ± 1.40 pg/mL/10^5^ cells in normal ductal cells, respectively (*n* = 3). The data indicated that the secretion of insulin was significantly decreased in differentiated pancreatic ductal cells derived from passage 15 (*P* < 0.05).

## Discussion

Diabetes mellitus (DM) is a common disease of dogs in internal medicine, and type 1 DM is the most common disease among dogs (1, 16). Type 1 DM, also known as insulin-dependent DM, results in the destruction of beta cells in pancreatic islets by the autoimmune system. Therefore, the replacement of destroyed beta cells with normal cells or the neogenesis of pancreatic beta cells from stem cells in the pancreas is an ideal therapy for type 1 DM. Pancreatic ductal cells are an important component of the pancreatic exocrine system, which expresses major markers of epithelial cells, such as CK19 in humans, mice, rats, rabbits, and other organisms (11). Many studies have reported that ductal cells can differentiate into insulin-secreting cells under special conditions, such as the ectopic expression of NGN3, Pax4, and Neuro D (17), deletion of specific genes (Fbw7) (18), and pancreatic injury (ductal ligation) (19, 20). Moreover, pancreatic ductal cells not only have differentiation potential but also express PDX1. PDX 1 is also known as islet/duodenum homeobox 1 (IDX1) or insulin promoter factor 1 (Ipf1), which is expressed in the foregut endoderm primarily, which later becomes the duodenum and pancreas, and then is uniformly expressed in pancreatic buds (21). However, a lack of PDX1 in the pancreas leads to selective agenesis and a reduction in glucose-stimulated insulin release in humans and mice (22, 23). These means that PDX1 not only regulates cell differentiation during pancreatic development but also influences glucose-stimulated insulin release in normal pancreatic beta cells. Our data revealed that the biological characteristics of pancreatic ductal cells from canines are similar to those of humans and mice. Therefore, pancreatic beta cell neogenesis from pancreatic ductal cells is an attractive strategy and ideal cell therapy for type 1 DM in canines.

One important property of pancreatic ductal cells is their ability to differentiate into beta cells (24, 25). HGF has multiple functions, including the activation of tissue regeneration, promotion of angiogenesis, inhibition of fibrosis, and promotion of stem cell differentiation by activating MAPK/ERK1/2 and PI3K/AKT signaling in mice and rats (26). Activins are dimeric proteins composed of either two βA subunits (activin A), two βB subunits (activin B), or a βA and βB subunit (activin AB). The activin-A-activated pathway is a part of the TGF-β pathway, and activin A is extensively used in the formation of pancreatic beta cells or beta-like cells from adult stem cells, embryonic stem cells, or induced pluripotent stem cells (13, 27–29). Moreover, the underlying mechanism involves activation of the Smad pathway to enhance transcription of pancreatic specific genes (*Neurog3*, miR-375, and miR-26a) to induce the formation of pancreatic beta cells from stem cells (29). Exendin-4 is also a frequently used factor in the fromation of pancreatic beta cells from adult stem cells, embryonic stem cells, or induced pluripotent stem cells, which induces the production of pancreatic beta cells (30). Nicotinamide, a poly (ADP-ribose) synthetase inhibitor, has been reported to prevent the development of diabetes in mice, promote the differentiation of pancreatic beta cells from stem cells, and increase their proliferation (31, 32). The aforementioned factors, HGF, activin-A, exendin-4, and nicotinamide, were added into the medium, and cells were cultured for 14 days. These cells not only expressed insulin and glucagon but also secreted insulin under high glucose treatment, indicating their successful differentiation into pancreatic beta cells.

Another important property of stem cells is their ability to self-renew. Pancreatic ductal cells from canines were cultured for at least 18 passages and maintained in culture *in vitro* for over 6 months in our study. However, the differentiation efficiency of beta cells dramatically decreased after 15 passages of pancreatic ductal cells from canines. Although the components of the cultured medium was suitable to induce the proliferation of pancreatic ductal cells, cells from higher passages lose their multi-lineage differentiation potential; therefore, the maintenance of self-renewal in pancreatic ductal cells from canines *in vitro* should be further investigated in the future.

In conclusion, pancreatic ductal cells from canines were isolated from pancreatic tissue, and their self-renewal ability and differentiation potential were evaluated in this study. Our results indicated that a mixture of HGF, activin-A, exendin-4, and nicotinamide can activate a series of intracellular signals to induce the production of islet beta cells from pancreatic ductal cells. Our study might assist in the development of effective cell therapies for the treatment of DM in dogs.

## Data Availability Statement

The original contributions presented in the study are included in the article/supplementary material, further inquiries can be directed to the corresponding author/s.

## Ethics Statement

The animal study was reviewed and approved by the Institutional Animal Care and Use Committee of the Chinese Academy of Agricultural Sciences and Jining Medical University. Written informed consent was obtained from the owners for the participation of their animals in this study.

## Author Contributions

YG performed cell culture, glucose-stimulated insulin secretion and FCM, and drafted the manuscript. CB performed production of beta-like islet cells and IF. WG provided the tissue of dog and participated in its coordination. CB and WG designed the experiments and reviewed the manuscript. All authors contributed to the article and approved the submitted version.

## Funding

This research was supported by the National Natural Science Foundation of China (Grant No. 31972755 to CB), Shandong Provincial Natural Science Foundation, China (Grant No. ZR2020KH031 to CB), Project of Shandong Province Higher Educational Youth Innovation Science and Technology Program (2019KJK010), Supporting Fund for Teachers' Research of Jining Medical University (JYFC2018KJ060 to YG), and Faculty Start-up Funds of Jining Medical University (CB and YG).

## Conflict of Interest

The authors declare that the research was conducted in the absence of any commercial or financial relationships that could be construed as a potential conflict of interest.

## Publisher's Note

All claims expressed in this article are solely those of the authors and do not necessarily represent those of their affiliated organizations, or those of the publisher, the editors and the reviewers. Any product that may be evaluated in this article, or claim that may be made by its manufacturer, is not guaranteed or endorsed by the publisher.

## References

[1] Nelson RW Reusch CE. Animal models of disease: classification and etiology of diabetes in dogs and cats. J Endocrinol. (2014) 222:T1–9. 10.1530/JOE-14-0202 24982466

[2] O'Kell AL Garrett TJ Wasserfall C Atkinson MA. Untargeted metabolomic analysis in naturally occurring canine diabetes mellitus identifies similarities to human type 1 diabetes. Sci Rep. (2017) 7:9467. 10.1038/s41598-017-09908-5 28842637 PMC5573354

[3] Grapin-Botton A. Ductal cells of the pancreas. Int J Biochem Cell Biol. (2005) 37:504–10. 10.1016/j.biocel.2004.07.010 15618005

[4] Jawahar AP Narayanan S Loganathan G Pradeep J Vitale GC Jones CM. Ductal cell reprogramming to insulin-producing beta-like cells as a potential beta cell replacement source for chronic pancreatitis. Curr Stem Cell Res Ther. (2019) 14:65–74. 10.2174/1574888X13666180918092729 30227823

[5] Rezania A Bruin JE Arora P Rubin A Batushansky I Asadi A. Reversal of diabetes with insulin-producing cells derived *in vitro* from human pluripotent stem cells. Nat Biotechnol. (2014) 32:1121–33. 10.1038/nbt.3033 25211370

[6] Russ HA Parent AV Ringler JJ Hennings TG Nair GG Shveygert M. Controlled induction of human pancreatic progenitors produces functional beta-like cells *in vitro*. EMBO J. (2015) 34:1759–72. 10.15252/embj.201591058 25908839 PMC4516429

[7] Broutier L Andersson-Rolf A Hindley CJ Boj SF Clevers H Koo BK. Culture and establishment of self-renewing human and mouse adult liver and pancreas 3D organoids and their genetic manipulation. Nat Protoc. (2016) 11:1724–43. 10.1038/nprot.2016.097 27560176

[8] Millman JR Xie C Van Dervort A Gurtler M Pagliuca FW Melton DA. Generation of stem cell-derived beta-cells from patients with type 1 diabetes. Nat Commun. (2016) 7:11463. 10.1038/ncomms11463 27163171 PMC4866045

[9] Qadir MMF Alvarez-Cubela S Klein D Van Dijk J Muniz-Anquela R Moreno-Hernandez YB. Single-cell resolution analysis of the human pancreatic ductal progenitor cell niche. Proc Natl Acad Sci USA. (2020) 117:10876–87. 10.1073/pnas.1918314117 32354994 PMC7245071

[10] Bai C Hou L Zhang M Pu Y Guan W Ma Y. Characterization of vascular endothelial progenitor cells from chicken bone marrow. BMC Vet Res. (2012) 8:54. 10.1186/1746-6148-8-54 22584105 PMC3408357

[11] Zulewski H Abraham EJ Gerlach MJ Daniel PB Moritz W Muller B. Multipotential nestin-positive stem cells isolated from adult pancreatic islets differentiate ex vivo into pancreatic endocrine, exocrine, and hepatic phenotypes. Diabetes. (2001) 50:521–33. 10.2337/diabetes.50.3.521 11246871

[12] Pennarossa G Maffei S Campagnol M Tarantini L Gandolfi F Brevini TA. Brief demethylation step allows the conversion of adult human skin fibroblasts into insulin-secreting cells. Proc Natl Acad Sci USA. (2013) 110:8948–53. 10.1073/pnas.1220637110 23696663 PMC3670366

[13] Pagliuca FW Millman JR Gurtler M Segel M Van Dervort A Ryu JH. Generation of functional human pancreatic beta cells *in vitro*. Cell. (2014) 159:428–39. 10.1016/j.cell.2014.09.040 25303535 PMC4617632

[14] Deconinck JF Potvliege PR Gepts W. The ultrasturcture of the human pancreatic islets. I. The islets of adults. Diabetologia. (1971) 7:266–82. 10.1007/BF01211879 4106080

[15] Like AA Orci L. Embryogenesis of the human pancreatic islets: a light and electron microscopic study. Diabetes. (1972) 21:511–34. 10.2337/diab.21.2.S511 4559917

[16] Rand JS Fleeman LM Farrow HA Appleton DJ Lederer R. Canine and feline diabetes mellitus: nature or nurture? J Nutr. (2004) 134:2072–80S. 10.1093/jn/134.8.2072S 15284406

[17] Noguchi H Xu G Matsumoto S Kaneto H Kobayashi N Bonner-Weir S. Induction of pancreatic stem/progenitor cells into insulin-producing cells by adenoviral-mediated gene transfer technology. Cell Transplant. (2006) 15:929–38. 10.3727/000000006783981431 17299998

[18] Sancho R Gruber R Gu G Behrens A. Loss of Fbw7 reprograms adult pancreatic ductal cells into alpha, delta, and beta cells. Cell Stem Cell. (2014) 15:139–53. 10.1016/j.stem.2014.06.019 25105579 PMC4136739

[19] Bonner-Weir S Inada A Yatoh S Li WC Aye T Toschi E. Transdifferentiation of pancreatic ductal cells to endocrine beta-cells. Biochem Soc Trans. (2008) 36:353–6. 10.1042/BST0360353 18481956

[20] Inada A Nienaber C Katsuta H Fujitani Y Levine J Morita R. Carbonic anhydrase II-positive pancreatic cells are progenitors for both endocrine and exocrine pancreas after birth. Proc Natl Acad Sci USA. (2008) 105:19915–9. 10.1073/pnas.0805803105 19052237 PMC2604974

[21] Ohlsson H Karlsson K Edlund T. IPF1, a homeodomain-containing transactivator of the insulin gene. EMBO J. (1993) 12:4251–9. 10.1002/j.1460-2075.1993.tb06109.x 7901001 PMC413720

[22] Jonsson J Carlsson L Edlund T Edlund H. Insulin-promoter-factor 1 is required for pancreas development in mice. Nature. (1994) 371:606–9. 10.1038/371606a0 7935793

[23] Stoffers DA Zinkin NT Stanojevic V Clarke WL Habener JF. Pancreatic agenesis attributable to a single nucleotide deletion in the human IPF1 gene coding sequence. Nat Genet. (1997) 15:106–10. 10.1038/ng0197-106 8988180

[24] Loomans CJM Williams Giuliani N Balak J Ringnalda F Van Gurp L Huch M. Expansion of adult human pancreatic tissue yields organoids harboring progenitor cells with endocrine differentiation potential. Stem Cell Rep. (2018) 10:712–24. 10.1016/j.stemcr.2018.02.005 29539434 PMC5918840

[25] Georgakopoulos N Prior N Angres B Mastrogiovanni G Cagan A Harrison D. Long-term expansion, genomic stability and *in vivo* safety of adult human pancreas organoids. BMC Dev Biol. (2020) 20:4. 10.1186/s12861-020-0209-5 32098630 PMC7043048

[26] Han P Cui Q Lu W Yang S Shi M Li Z. Hepatocyte growth factor plays a dual role in tendon-derived stem cell proliferation, migration, and differentiation. J Cell Physiol. (2019) 234:17382–91. 10.1002/jcp.28360 30807656

[27] Shi Y Hou L Tang F Jiang W Wang P Ding M. Inducing embryonic stem cells to differentiate into pancreatic beta cells by a novel three-step approach with activin A and all-trans retinoic acid. Stem Cells. (2005) 23:656–62. 10.1634/stemcells.2004-0241 15849173

[28] Bai C Gao Y Li X Wang K Xiong H Shan Z. MicroRNAs can effectively induce formation of insulin-producing cells from mesenchymal stem cells. J Tissue Eng Regen Med. (2017) 11:3457–68. 10.1002/term.2259 28397402

[29] Gao Y Zhang R Dai S Zhang X Li X Bai C. Role of TGF-beta/Smad pathway in the transcription of pancreas-specific genes during beta cell differentiation. Front Cell Dev Biol. (2019) 7:351. 10.3389/fcell.2019.00351 31921861 PMC6933421

[30] Kassem DH Kamal MM El-Kholy Ael L El-Mesallamy HO. Exendin-4 enhances the differentiation of Wharton's jelly mesenchymal stem cells into insulin-producing cells through activation of various beta-cell markers. Stem Cell Res Ther. (2016) 7:108. 10.1186/s13287-016-0374-4 27515427 PMC4981957

[31] Uchigata Y Yamamoto H Nagai H Okamoto H. Effect of poly(ADP-ribose) synthetase inhibitor administration to rats before and after injection of alloxan and streptozotocin on islet proinsulin synthesis. Diabetes. (1983) 32:316–8. 10.2337/diab.32.4.316 6299867

[32] Otonkoski T Beattie GM Mally MI Ricordi C Hayek A. Nicotinamide is a potent inducer of endocrine differentiation in cultured human fetal pancreatic cells. J Clin Invest. (1993) 92:1459–66. 10.1172/JCI116723 8104197 PMC288291

